# COL1A1, ITGB1, THY1, and PDGFRA: key immune-related genes in uterine corpus endometrial carcinoma with prognostic and therapeutic implications

**DOI:** 10.1186/s41065-025-00448-x

**Published:** 2025-08-15

**Authors:** Taghreed N. Almanaa, Abdulaziz Alamri, Mostafa A. Abdel-Maksoud, Ibrahim A. Saleh, Naser Zomot, Jehad S. Al-Hawadi, Wahidah H. Al-Qahtani, Yasir Hameed

**Affiliations:** 1 Department of Botany and Microbiology, College of Science, King Saud University, Riyadh, 11451 Saudi Arabia; 2 Biochemistry Department, College of Science, King Saud University, Riyadh, Saudi Arabia; 3 Chair of Biomedical Applications of Nanomaterials, Biochemistry Department-College of Science-King Saud University, P.O. Box 2455, Riyadh, 11451 Saudi Arabia; 4 Faculty of Science, Zarqa University, Zarqa, 13110 Jordan; 5 Department of Food Sciences & Nutrition, College of Food and Agricultural Sciences, King Saud University, P.O. Box 270677, Riyadh, 11352 Saudi Arabia; 6 Department of Biochemistry and Biotechnology, The Islamia University of Bahawalpur, Bahawalpur, 63100 Pakistan; 7 Department of Applied Biological Sciences, Tokyo University of Science, Tokyo, Japan

**Keywords:** Immune-related genes, UCEC, Diagnosis, Prognosis, Treatment

## Abstract

Uterine corpus endometrial carcinoma (UCEC) is one of the most common gynecological malignancies, characterized by complex molecular alterations that drive its progression. Understanding the molecular mechanisms underlying UCEC is crucial for developing effective diagnostic, prognostic, and therapeutic strategies. Immune-related genes, such as COL1A1, ITGB1, THY1, and PDGFRA, have been implicated in various cancers, but their roles in UCEC remain underexplored. In this study, we investigate the roles of these genes in the development and progression of UCEC. Using both in silico and in vitro approaches, we found that these genes were dysregulated in UCEC. Our results revealed the downregulation of COL1A1, ITGB1, THY1, and PDGFRA in UCEC compared to normal tissues. Further, promoter methylation analysis showed increased methylation of these genes in UCEC. Survival analysis highlighted their potential as prognostic markers, with lower expression linked to poor patient survival. Additionally, genetic alteration analysis demonstrated mutations in these genes across UCEC patients. Our results also showed that overexpression of COL1A1 in KLE and HEC-1B cells significantly reduced cell proliferation, colony formation, and migration, indicating that COL1A1 overexpression impacts critical cellular behaviors in UCEC. Finally, we explored the therapeutic potential of targeting these genes, suggesting that they may offer valuable insights for personalized treatment strategies in UCEC. This study identifies COL1A1, ITGB1, THY1, and PDGFRA as crucial regulators of UCEC progression, with altered expression linked to tumor behavior and patient survival. Overexpression of COL1A1 impaired cell proliferation, colony formation, and migration. Future research should focus on elucidating the molecular mechanisms of these genes, exploring their therapeutic targeting in preclinical models, and validating their clinical potential as biomarkers in larger patient cohorts to improve treatment strategies for UCEC.

**Supplementary Information:**

The online version contains supplementary material available at 10.1186/s41065-025-00448-x.

## Introduction

Uterine corpus endometrial carcinoma (UCEC) is one of the most prevalent gynecological malignancies worldwide, accounting for a significant burden of morbidity and mortality among women [1]. UCEC predominantly affects postmenopausal women, with risk factors including obesity, hormone imbalance, and genetic predispositions such as Lynch syndrome [1, 2]. Despite advancements in diagnostic tools and therapeutic approaches, including surgery, radiation, and hormone therapy, the incidence and mortality rates of UCEC have been steadily rising in recent years [3, 4]. This concerning trend can be attributed, in part, to the increasing prevalence of risk factors like obesity, metabolic syndrome, and aging populations, along with late-stage diagnoses and the limited efficacy of conventional treatments in advanced or recurrent cases [5]. This increased prevalence of UCEC emphasizes the urgent need for a deeper understanding of the molecular mechanisms underlying UCEC pathogenesis and progression.

In the current study, we investigate the roles of four genes—COL1A1, ITGB1, THY1, and PDGFRA—in shaping the immune landscape of UCEC, using in silico and in vitro approaches. These genes, known for their roles in tissue remodeling, cell migration, and immune interactions, have been implicated in immune evasion in various cancers.

COL1A1 encodes collagen type I, which supports tissue structure and promotes immune suppression by creating a dense extracellular matrix (ECM) that inhibits immune cell infiltration [6]. In cancers such as breast and pancreatic cancer, COL1A1 overexpression has been associated with a desmoplastic reaction that fosters tumor growth and hampers immune responses [7]. ITGB1 regulates immune cell adhesion and migration by mediating interactions with the ECM. In cancers like breast and pancreatic cancer, dysregulation of ITGB1 has been shown to promote immune evasion by modulating immune cell trafficking and facilitating tumor invasion [8]. THY1 (CD90) is involved in immune cell activation and interaction with fibroblasts, contributing to immune regulation and inflammation [9]. In melanoma and glioblastoma, THY1 overexpression enhances tumor progression and immune suppression by activating cancer-associated fibroblasts (CAFs) and altering immune cell behavior in the tumor microenvironment [10, 11]. PDGFRA encodes a receptor that controls cell proliferation and fibroblast activation, playing a key role in tumor-associated fibrosis [12]. In cancers such as gastrointestinal stromal tumors (GIST) and gliomas, PDGFRA-driven fibrosis creates an immune-suppressive environment that limits immune cell infiltration and promotes tumor survival [13].

In this comprehensive study, we aim to explore the role of these critical immune-related genes in the pathogenesis and progression of UCEC. We will investigate the molecular functions, mechanisms of dysregulation, and clinical implications of COL1A1, ITGB1, THY1, and PDGFRA in UCEC using a combination of in silico analyses and molecular experiments. Additionally, we will explore the therapeutic potential of targeting these genes and their associated pathways in UCEC.

## Methodology

### Cell culture

15 UCEC cell lines (KLE, Ishikawa, RL95-2, SNG-M, USPC-2, MFE-280, SPAC-1-L, SPAC-1-S, EN, UU, MFE-296, MFE-319, SK-UT-1, SK-UT-1B, and SPAC-1-N) and 15 normal endometrial cell lines (HEMn-LP, HCv, NCx-2, NEC-1, HCx-4, NCx-3, NCx-4, NEC-2, NCx-5, HCv-2, HCv-3, HCv-4, NEC-3, NEC-4, and NCx-6) were sourced from the American Type Culture Collection (ATCC, Manassas, VA, USA). All cell lines were cultured in RPMI 1640 medium (Invitrogen, Carlsbad, CA, USA) supplemented with 10% fetal bovine serum (FBS, Gibco, California, USA) and 1% antibiotics solution (Corning, New York, USA) at 37 °C in a 5% CO_2_ atmosphere.

### Reverse transcription quantitative polymerase chain reaction (RT-qPCR) analysis of COL1A1, ITGB1, THY1, and PDGFRA

Total RNA was extracted from cells utilizing the RNA fast200 Extraction kit (Fastagen Biotech, China), followed by quantification of RNA concentration using a NanoDrop 2000 spectrophotometer (Thermo, United States). Subsequently, reverse transcription was performed using the PrimeScript RT Master Mix (Takara, Japan), followed by qRT-PCR analysis using Taq Pro Universal SYBR qPCR Master Mix (Vazyme, China) on the CFX96 Real-Time System (Bio-Rad, United States). GAPDH was employed as the internal control for normalization. Primers were procured from Sangon Biotech (Shanghai, China), and their sequences are provided below:

GAPDH-F 5’-ACCCACTCCTCCACCTTTGAC-3’,

GAPDH-R 5’-CTGTTGCTGTAGCCAAATTCG-3’.

COL1A1-F: 5’-GATTCCCTGGACCTAAAGGTGC-3’.

COL1A1-R: 5’-AGCCTCTCCATCTTTGCCAGCA-3’.

ITGB1-F: 5’-GGATTCTCCAGAAGGTGGTTTCG-3’.

ITGB1-R: 5’-TGCCACCAAGTTTCCCATCTCC-3’.

THY1-F: 5’-GAAGGTCCTCTACTTATCCGCC-3’.

THY1-R: 5’-TGATGCCCTCACACTTGACCAG-3’.

PDGFRA-F: 5’-GCAGTTGCCTTACGACTCCAGA-3’.

PDGFRA-R: 5’-GCAGTTGCCTTACGACTCCAGA-3’.

### Receiver operating characteristic (ROC)

ROC curves based on expression and promoter methylation were employed to assess the potential of COL1A1, ITGB1, THY1, and PDGFRA genes as diagnostic biomarkers for detecting UCEC.

### Validation of COL1A1, ITGB1, THY1, and PDGFRA mRNA and protein expression using pooled TCGA and HPA datasets

The UALCAN database provides a user-friendly platform for analyzing cancer transcriptome data from The Cancer Genome Atlas (TCGA). It enables researchers to explore gene expression profiles, clinical data, and survival information across various cancer types [14]. GEPIA2, on the other hand, offers comprehensive gene expression analysis based on TCGA and Genotype-Tissue Expression (GTEx) data, allowing users to compare gene expression between tumor and normal tissues [15, 16]. Additionally, the Human Protein Atlas (HPA) database provides valuable insights into protein expression profiles in normal and cancerous tissues through immunohistochemistry-based assays, facilitating the study of protein localization and function in cancer biology [17, 18]. In the current work, UALCAN and GEPIA2 were used to validate mRNA expression levels while the HPA database was used to validate protein expression levels of COL1A1, ITGB1, THY1, and PDGFRA across UCEC patients.

### Promoter methylation analysis of COL1A1, ITGB1, THY1, and PDGFRA

The OncoDB database is a valuable resource for cancer researchers, providing comprehensive information on DNA methylation patterns, gene expression profiles, and genomic alterations across various cancer types [19]. It integrates data from large-scale cancer genomics studies, such as The Cancer Genome Atlas (TCGA), allowing users to explore the molecular landscape of cancer and identify potential biomarkers and therapeutic targets. In this work, this database was used to analyze promoter methylation levels of COL1A1, ITGB1, THY1, and PDGFRA in UCEC.

### Survival analysis

The KM Plotter serves as a crucial online tool for survival analysis within cancer research [20]. This resource enables researchers to evaluate the influence of individual genes on patient survival outcomes across various cancer types. In our study, we used the KM Plotter platform to conduct a survival analysis of COL1A1, ITGB1, THY1, and PDGFRA in UCEC patients.

### Genetic alteration analysis

The cBioPortal platform was utilized to investigate mutation features of COL1A1, ITGB1, THY1, and PDGFRA across UCEC, leveraging its extensive cancer genomics data [21]. Through the “quick selection” module, these genes were analyzed for genetic alterations, and their frequencies were assessed using the “Cancer Types Summary” module. Detailed mutation sites and three-dimensional structures were obtained using the “Mutations” module. Additionally, waterfall plots illustrating the genetic alterations of these genes were generated via the “OncoPrint” module, providing a comprehensive understanding of their mutational landscape in UCEC.

### miRNA prediction and validation

ENCORI database is a comprehensive platform for exploring non-coding RNA (ncRNA) interactions in cancer [22]. It integrates multiple datasets and provides tools for analyzing RNA expression, regulation, and interactions with miRNAs, lncRNAs, and circRNAs. ENCORI facilitates research into the role of ncRNAs in cancer biology and therapy development. In the present study, ENCORI database was used to predict COL1A1, ITGB1, THY1, and PDGFRA-associated miRNAs.

Later on, the expression of two valuable miRNAs, including has-mir-16-5p and has-mir-128-3p was analyzed in clinical UCEC samples via RT-qPCR using aftermentioned protocol. Primers were procured from Sangon Biotech (Shanghai, China), and their sequences are provided below:

RNU6-F 5′-CTCGCTTCGGCAGCACA-3′.

RNU6-R 5′-AACGCTTCACGAATTTG-3′.

has-mir-16-5p 5′-TAGCAGCACGTAAATATTGGCG-3′.

has-mir-128-3p-F 5’-UCACAGUGAACCGGUCUCUUU-3′.

has-mir-128-3p-R 5’-AGAGACCGGUUCACUGUGAUU-3’.

### Immune correlation analysis

TIMER2.0 is a comprehensive web tool offering valuable insights into immune infiltration patterns across various cancer types [23]. This resource harnesses transcriptomic data sourced from TCGA to quantify the presence of tumor-infiltrating immune cells and discern their clinical relevance. In our current study, we employed the TIMER2.0 database to assess the potential correlations existing between the expression levels of COL1A1, ITGB1, THY1, and PDGFRA and the extent of infiltration by distinct immune cell populations in UCEC.

### Gene enrichment analysis

To delve deeper into the roles of COL1A1, ITGB1, THY1, and PDGFRA, Gene Ontology (GO) and KEGG analyses were conducted using the DAVID tool [24]. These analyses aimed to elucidate the biological processes, molecular functions, cellular components, and pathways associated with these genes, providing insights into their functional significance and potential involvement in critical cellular mechanisms and signaling pathways relevant to cancer progression.

### Drug sensitivity analysis

Gene Set Enrichment Analysis (GSCA) stands as an extensive pharmacological repository, offering intricate insights into drugs and their corresponding targets [25]. By consolidating data on drug mechanisms, interactions, and potential adverse effects, GSCA facilitates researchers in the realms of drug discovery and development. In our current investigation, we utilized the GSCA database to conduct a meticulous analysis of drug sensitivity concerning the COL1A1, ITGB1, THY1, and PDGFRA genes.

### Inducing overexpression of COL1A1 in KLE and HEC-1B cells

To investigate the effects of COL1A1 overexpression, we transfected KLE and HEC-1B cells with expression vectors containing the COL1A1 gene. We utilized Thermo Fisher’s pcDNA3.1/V5-His-TOPO^®^ Vector, a versatile plasmid vector designed for high-level expression of target genes in mammalian cells. The COL1A1 gene was cloned into this plasmid backbone to create the expression construct. Cells were seeded into 6-well plates and grown until they reached 70–80% confluency. For the transfection process, we employed Thermo Fisher’s Lipofectamine™ 3000 Transfection Reagent, following the manufacturer’s instructions. The transfection reagent was mixed with the pcDNA3.1/COL1A1 plasmid DNA and incubated with the cells for 4–6 h. After this period, the transfection medium was replaced with fresh culture medium to allow for recovery and expression of the COL1A1 protein. Control cells were transfected with the empty pcDNA3.1/V5-His-TOPO^®^ Vector to serve as a baseline comparison.

Firstly, to assess the expression levels of COL1A1 in overexpressed and control cells, RT-qPCR was performed following aftermentioned conditions. For Western blot analysis, cells were lysed with RIPA buffer containing protease inhibitors, and protein concentration was measured using the Pierce BCA Protein Assay Kit. Equal protein amounts were separated on a 10% SDS-PAGE gel, transferred to PVDF membranes, and probed with primary antibodies against COL1A1 and GAPDH, followed by HRP-conjugated secondary antibodies. Protein bands were visualized using chemiluminescence (Thermo Fisher). The protocol followed was based on previous studies [26, 27].

### Colony formation assay

The colony formation assay was used to measure the proliferative and growth potential of KLE and HEC-1B cells. Cells were seeded at low density (500 cells per well) into 6-well plates and allowed to grow for 2–3 weeks to form colonies. After the incubation period, the cells were fixed with Thermo Fisher Formalin Solution (Catalog Number: 323926) for 10 min. Following fixation, the cells were stained with Thermo Fisher Crystal Violet Solution (Catalog Number: C0775) for 30 min. The wells were then rinsed with water and air-dried. Colony counts were performed to evaluate the colony formation efficiency.

### Cell proliferation assay

Cells overexpressing COL1A1 were seeded into a 96-well plate at a density of approximately 2,000 cells per well. The cells were allowed to adhere and grow in a humidified incubator at 37 °C and 5% CO_2_. Proliferation was measured at 24, 48, and 72 h by adding 10 µL of CCK-8 solution (from the CCK-8 kit, Thermo Fisher) to each well. The plate was incubated for 1–4 h at 37 °C, during which the CCK-8 reagent was metabolized by viable cells to form a water-soluble formazan product. The absorbance of each well was measured at 450 nm using a microplate reader. The absorbance value was directly proportional to the number of living cells, and cell proliferation was quantified by comparing the absorbance at each time point.

### Wound healing assay

The wound healing assay was conducted to evaluate cell migration and wound closure. Cells were seeded into 6-well plates and allowed to form a confluent monolayer. A wound was created by scratching the monolayer with a sterile pipette tip or a wound scratch tool. After removing cell debris by washing, fresh culture medium was added, and the cells were incubated at 37 °C in a CO_2_ incubator. Images of the wound area were captured at 0 h (initial) and 24 h using a microscope.

### Statistics

In this study, for Gene Ontology (GO) and Kyoto Encyclopedia of Genes and Genomes (KEGG) enrichment analysis, we employed the Fisher’s Exact test to compute the differences [28]. Correlational analyses were carried out using the Wilcoxon rank-sum and Pearson methods. A Student t-test was employed for making comparisons. All the analyses were conducted using R version 3.6.3 software. P-values < 0.05*, 0.01**, 0.001*** were considered as significant.

## Results

### Transcriptional expression level of COL1A1, ITGB1, THY1, and PDGFRA genes in UCEC and normal control cell lines

**Fig. 1:**
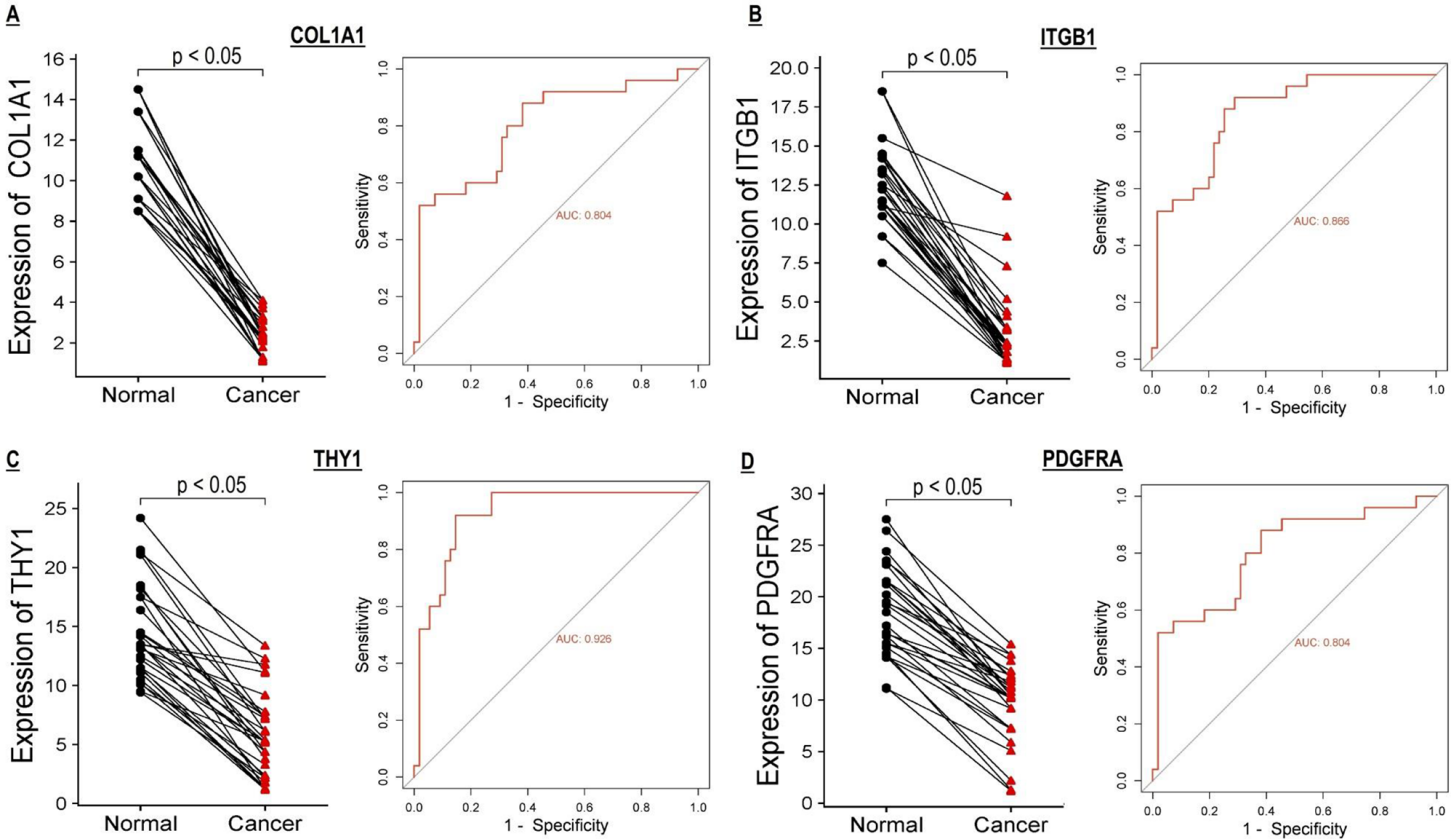
The expression profiles and receiver operating characteristic (ROC) curves of COL1A1, ITGB1, THY1, and PDGFRA. (**A**) Expression graph and ROC curve of COL1A1. (**B**) Expression graph and ROC curve of ITGB1. (**C**) Expression graph and ROC curve THY1. (**D**) Expression graph and ROC curve of PDGFRA. The statistical significance is indicated by a p-value < 0.05

To assess the transcriptional expression levels of COL1A1, ITGB1, THY1, and PDGFRA in UCEC cell lines (*n* = 15) versus corresponding normal control cell lines (*n* = 15), we performed RT-qPCR analysis. The results revealed a statistically significant (p-value < 0.05) down-regulation of COL1A1, ITGB1, THY1, and PDGFRA mRNA levels in UCEC cell lines compared to normal control cell lines (Fig. 1A-D). Additionally, ROC curve analysis based on transcriptional expression levels of these genes highlighted their potential diagnostic value for UCEC detection, although further validation with larger patient cohorts is necessary to confirm their clinical utility (Fig. 1A-D).

### Validation of COL1A1, ITGB1, THY1, and PDGFRA mRNA and protein expression using pooled TCGA and HPA datasets

**Fig. 2:**
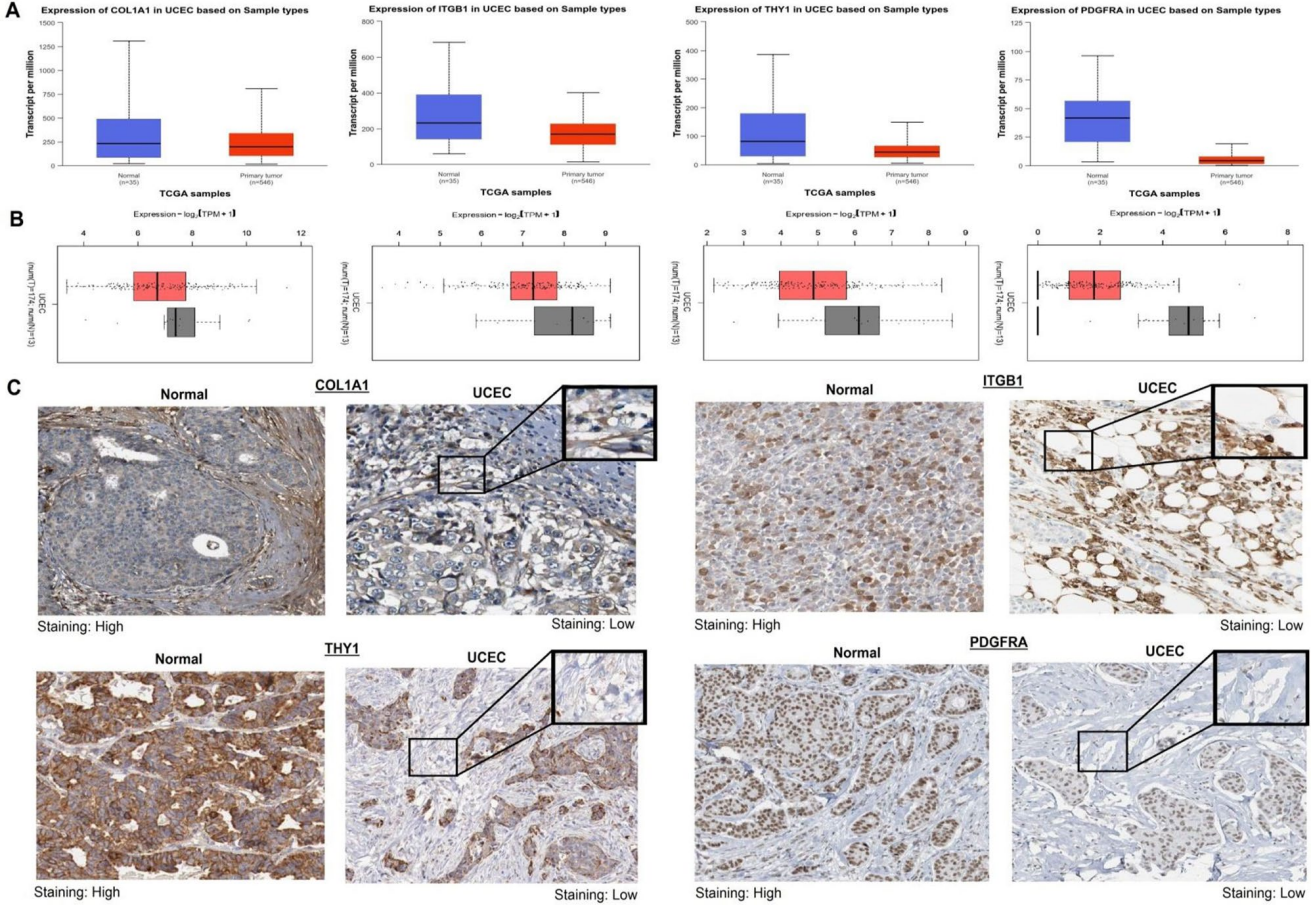
Validation of COL1A1, ITGB1, THY1, and PDGFRA expression levels in additional cohorts from The Cancer Genome Atlas (TCGA) and Human Protein Atlas databases. (**A**) It depicts mRNA level expression profiling of these genes in the Uterine Corpus Endometrial Carcinoma (UCEC) TCGA cohort through the UALCAN database. (**B**) The figure also shows mRNA level expression profiling of COL1A1, ITGB1, THY1, and PDGFRA in the UCEC TCGA cohort using the GEPIA2 database. (**C**) Additionally, the protein level expression profiling of these genes in the UCEC TCGA cohort is presented using the HPA database. The statistical significance is indicated by a p-value < 0.05

The transcriptional levels of COL1A1, ITGB1, THY1, and PDGFRA were evaluated in TCGA UCEC samples and normal control samples using the UALCAN and GEPIA databases. Consistent with the RT-qPCR results, the analysis revealed a significant down-regulation of COL1A1, ITGB1, THY1, and PDGFRA in UCEC samples compared to normal controls (p-value < 0.05) (Fig. 2A-B). Following the assessment of transcriptional levels, we further validated the protein expression levels of these genes in UCEC tissues using the HPA database. As shown in Fig. 2C, the protein expression of COL1A1, ITGB1, THY1, and PDGFRA was lower in UCEC tissues compared to normal control tissues, although further confirmation through experimental techniques would be valuable (Fig. 2C).

### Correlation of COL1A1, ITGB1, THY1, and PDGFRA genes with cancer stage and promoter methylation analysis

**Fig. 3:**
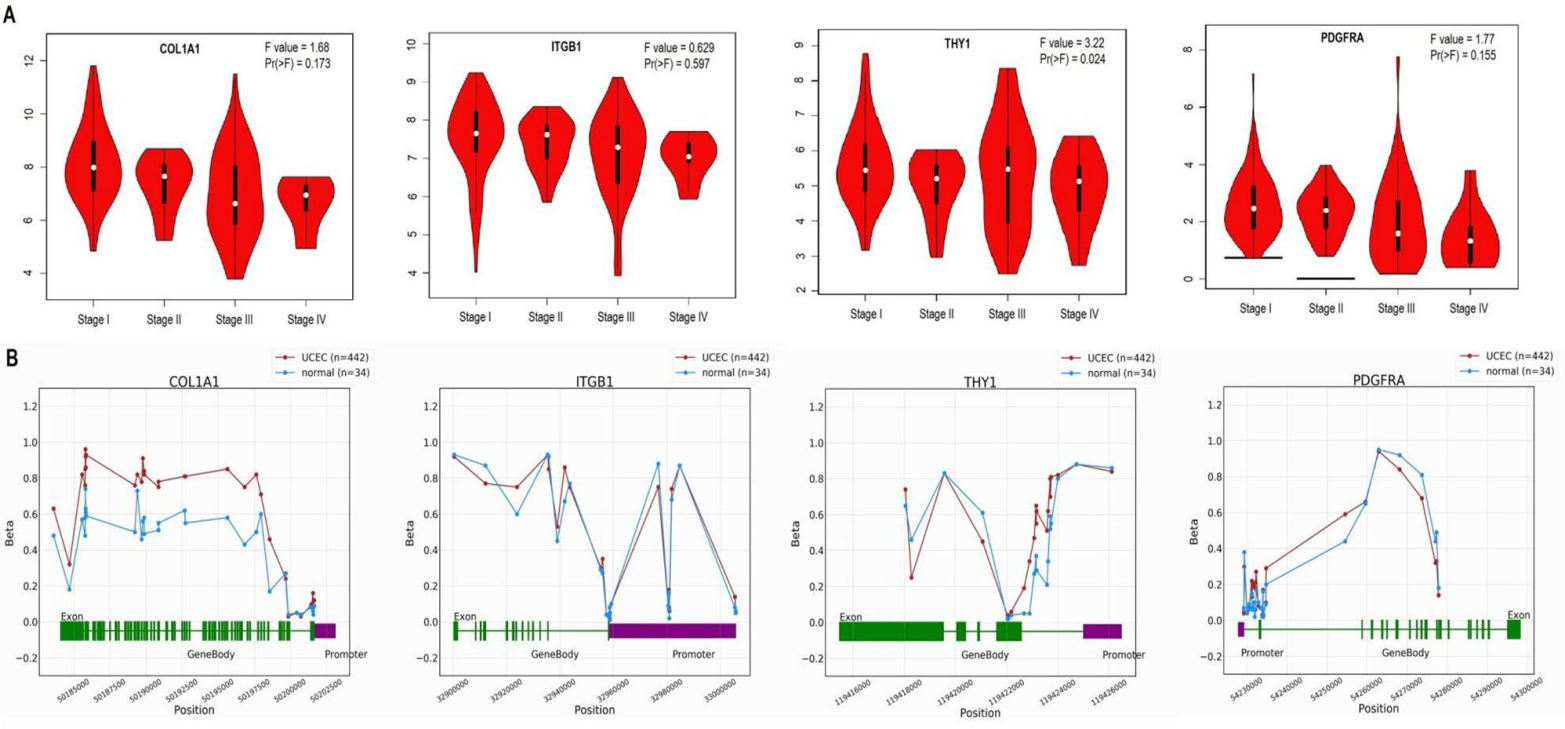
Analysis of COL1A1, ITGB1, THY1, and PDGFRA expression in different cancer stages and exploring promoter methylation levels in The Cancer Genome Atlas (TCGA) Uterine Corpus Endometrial Carcinoma (UCEC) cohorts using the GEPIA2 and OncoDB database. (**A**) Expression pattern of COL1A1, ITGB1, THY1, and PDGFRA in UCEC patients of different cancer stages. (**B**) Promoter methylation levels of COL1A1, ITGB1, THY1, and PDGFRA in UCEC and normal control samples. The statistical significance is indicated by a p-value < 0.05

To assess the clinical relevance of COL1A1, ITGB1, THY1, and PDGFRA genes in UCEC progression, we examined the correlation between their transcriptional expression levels and cancer stages. Our analysis revealed associations between the expression of COL1A1, ITGB1, THY1, and PDGFRA genes and different cancer stages in UCEC, however, the results were insignificant due to the lesser number of samples (Fig. 3A). To explore the potential relationship between COL1A1, ITGB1, THY1, and PDGFRA expression and DNA methylation, we analyzed the promoter methylation levels of these genes in UCEC tissues using the OncoDB database. Our findings revealed higher levels of methylation on the promoters of COL1A1, ITGB1, THY1, and PDGFRA in UCEC samples compared to normal tissues (p-value < 0.05) (Fig. 3B).

### Survival analysis

**Fig. 4:**
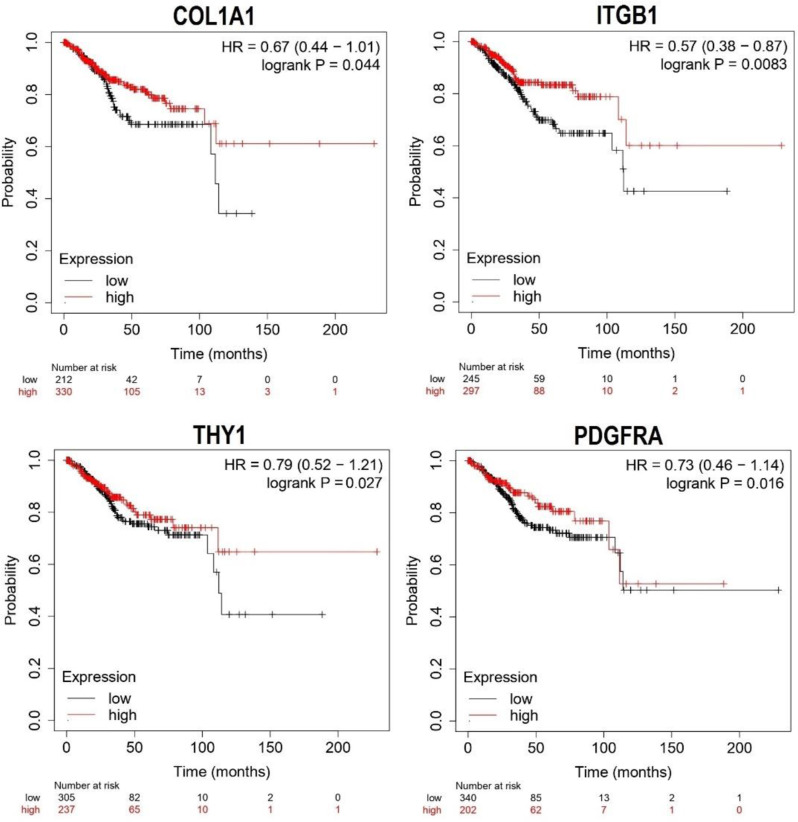
Survival analysis of the COL1A1, ITGB1, THY1, and PDGFRA genes in Uterine Corpus Endometrial Carcinoma (UCEC). Kaplan Meier (KM) curves of COL1A1, ITGB1, THY1, and PDGFRA genes in UCEC patients has been shown in this figure. The statistical significance is indicated by a p-value < 0.05

In this part of the study, our objective was to determine the prognostic relevance of COL1A1, ITGB1, THY1, and PDGFRA expression levels in UCEC. Kaplan-Meier survival curves from the KM plotter tool were utilized to examine the association between the expression of these genes and the survival outcomes of UCEC patients. Stratification based on median expression levels revealed that lower expression levels of COL1A1, ITGB1, THY1, and PDGFRA were significantly (p-value < 0.05) linked to poorer overall survival (OS) in UCEC patients (Fig. 4), highlighting their potential prognostic significance.

### Genetic alteration analysis

**Fig. 5:**
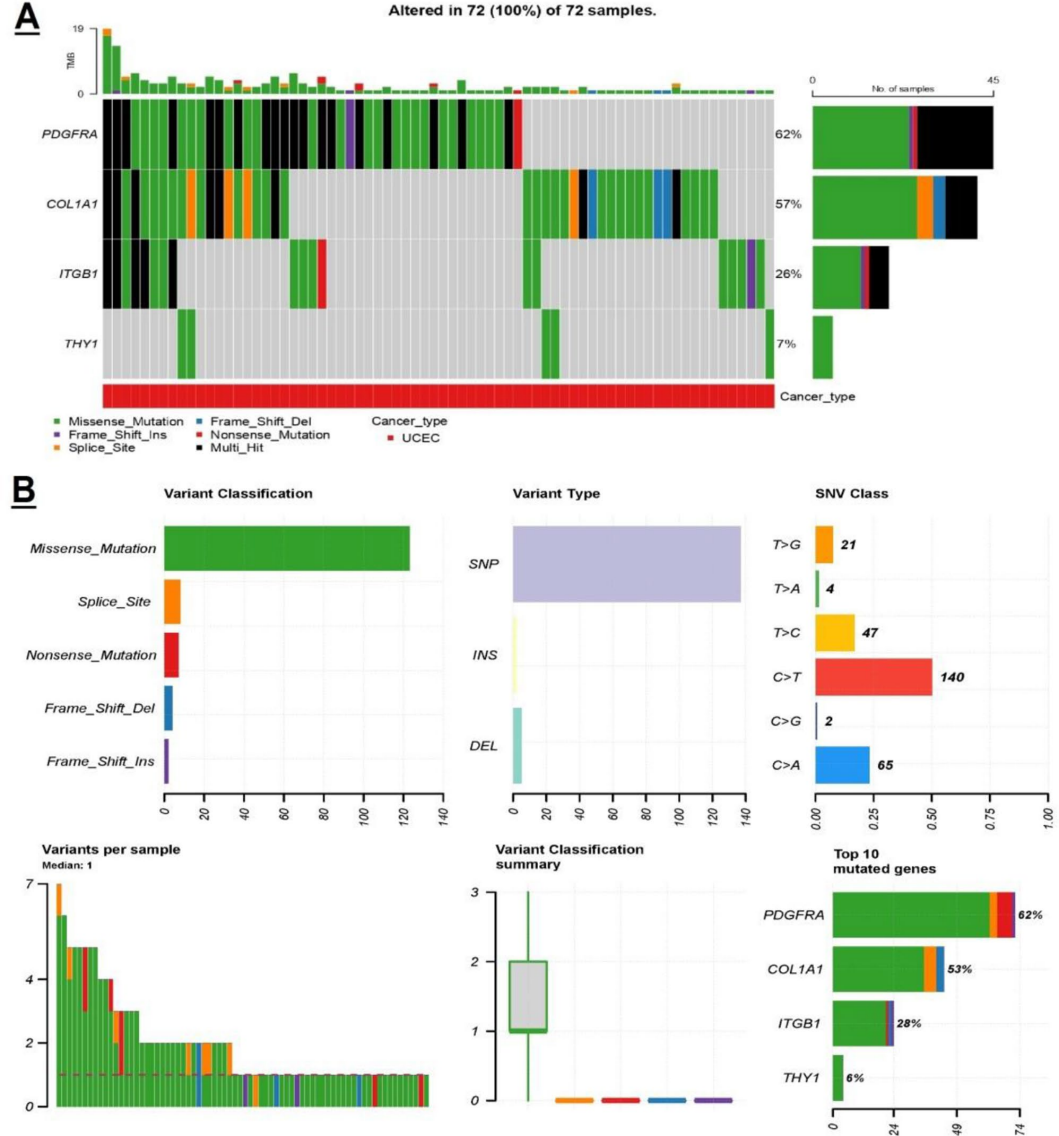
Mutational profiling of COL1A1, ITGB1, THY1, and PDGFRA genes across The Cancer Genome Atlas (TCGA) Uterine Corpus Endometrial Carcinoma (UCEC) cohorts using cBioPortal database. (**A**) Frequencies of the mutated UCEC samples. (**B**) Detail of the observed mutations

We conducted further analysis on the alterations of COL1A1, ITGB1, THY1, and PDGFRA genes in UCEC using the cBioPortal online tool. Our findings showed PDGFRA was altered in 62% of UCEC patients, followed by COL1A1 in 57%, ITGB1 in 26%, and THY1 in 7% of UCEC patients (Fig. 5A). Additionally, missense mutations were found to be the most common type of mutations observed in COL1A1, ITGB1, THY1, and PDGFRA across UCEC patients (Fig. 5B).

### miRNA prediction and validation

**Fig. 6:**
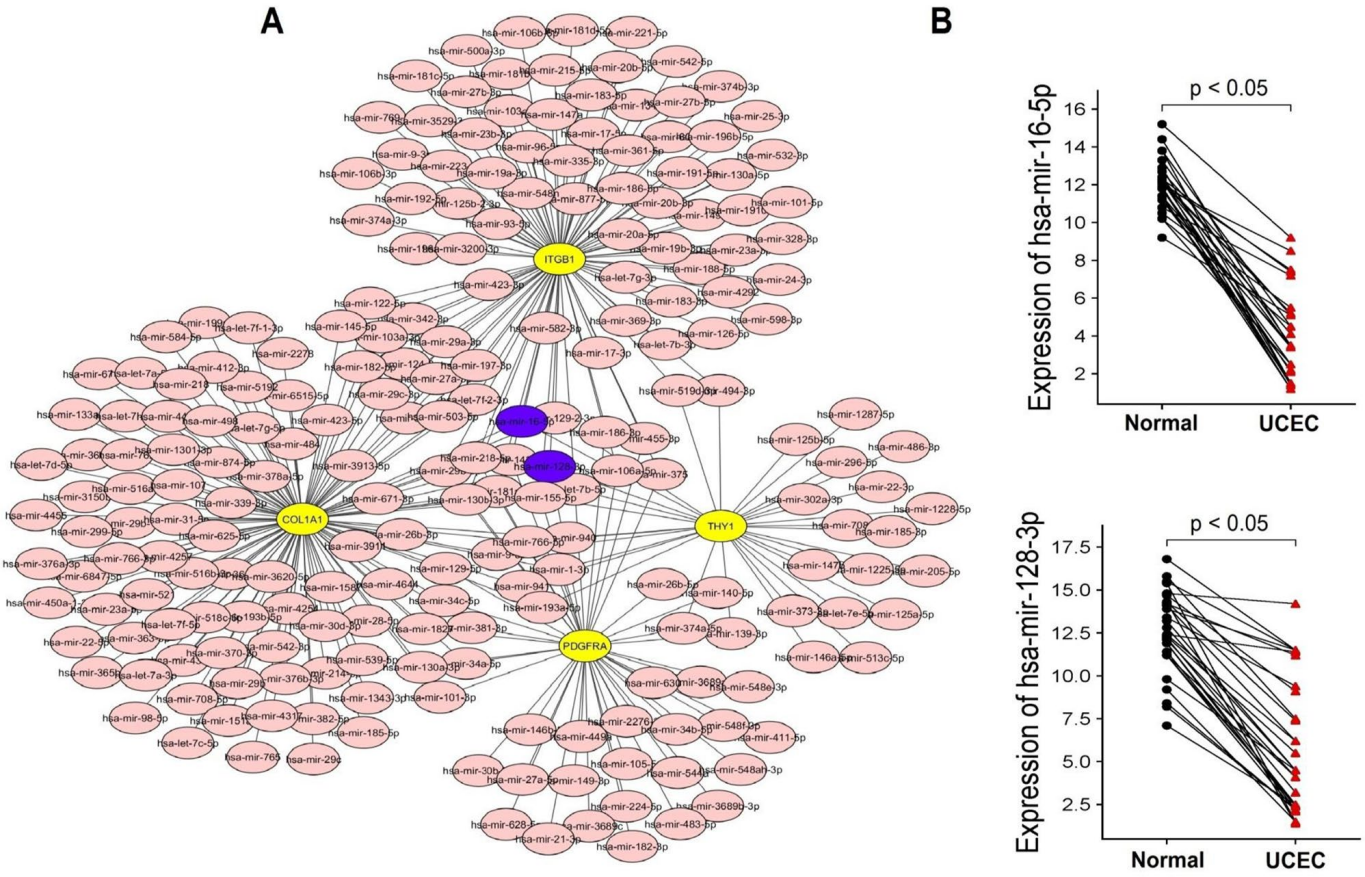
Prediction and validation of COL1A1, ITGB1, THY1, and PDGFRA genes-related miRNAs via the ENCORI database and RT-qPCR assay. (**A**) PPI networks of the COL1A1, ITGB1, THY1, and PDGFRA genes-targeting miRNAs. (**B**) Expression pattern of the has-mir-16-5p and has-mir-128-3p miRNAs in Uterine Corpus Endometrial Carcinoma (UCEC) and normal control cell lines. The statistical significance is indicated by a p-value < 0.05

To delve into the regulatory mechanisms governing the expression of COL1A1, ITGB1, THY1, and PDGFRA in UCEC, we utilized the ENCORI database to analyze miRNAs associated with these genes. Our analysis revealed 222 miRNAs implicated in regulating the expression of COL1A1, ITGB1, THY1, and PDGFRA in UCEC (Fig. 6A). Notably, among these miRNAs, two stood out as significant: has-mir-16-5p and has-mir-128-3p, both of which were found to regulate the expression of all four genes simultaneously in UCEC patients (Fig. 6A). Furthermore, we conducted RT-qPCR analysis to assess the expression patterns of has-mir-16-5p and has-mir-128-3p miRNAs in UCEC (*n* = 15) and normal control (*n* = 15) cell lines. The results revealed a significant down-regulation (p-value < 0.05) in the expression levels of both has-mir-16-5p and has-mir-128-3p miRNAs in UCEC cell lines compared to the control cell lines (Fig. 6B).

### Immune correlation analysis

**Fig. 7:**
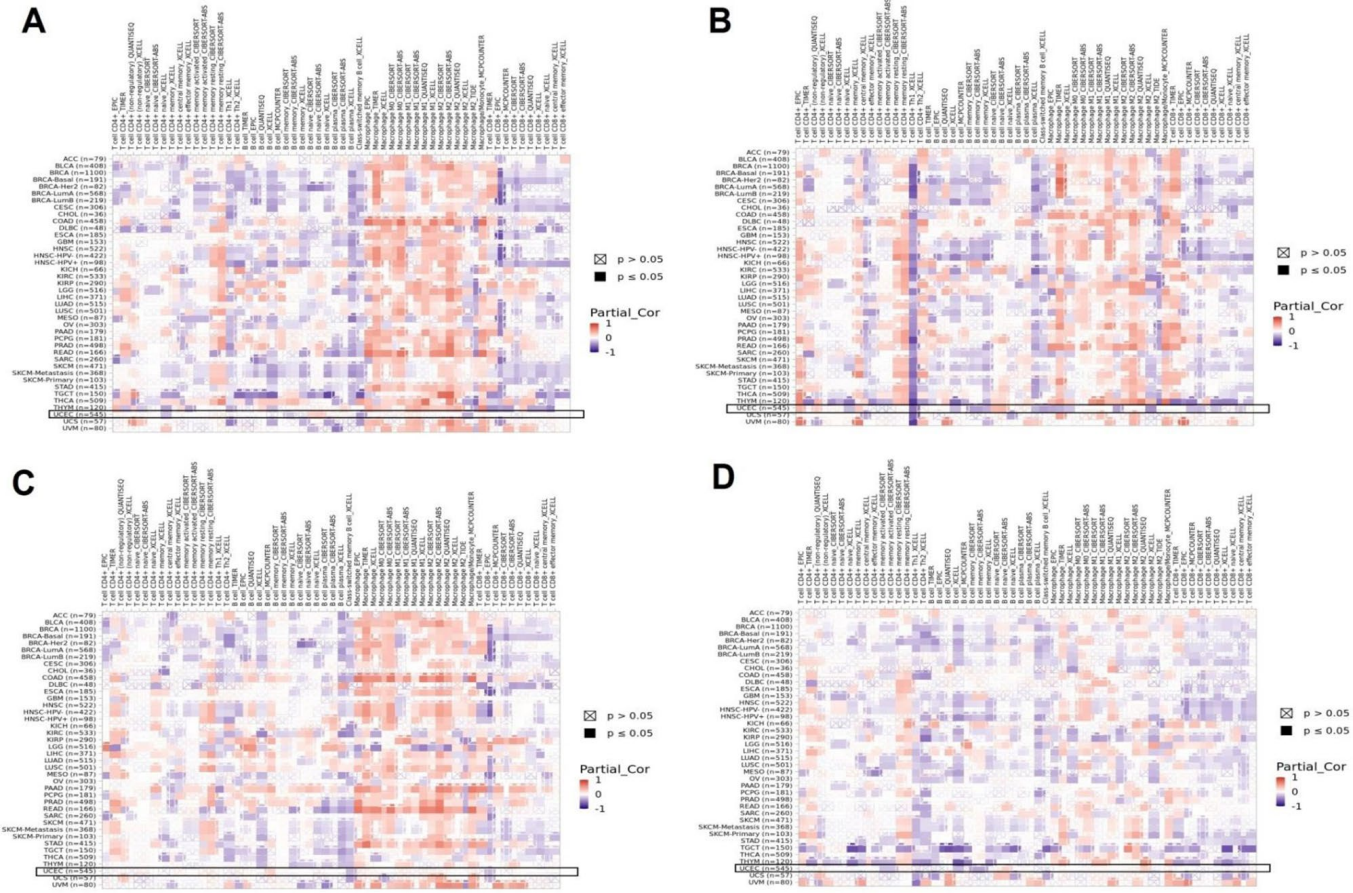
Correlation of COL1A1, ITGB1, THY1, and PDGFRA gene expression with the infiltration level of immune cells in Uterine Corpus Endometrial Carcinoma (UCEC). (**A**) Correlation of COL1A1 gene expression with the infiltration level of immune cells in UCEC. (**B**) Correlation of ITGB1 gene expression with the infiltration level of immune cells in UCEC. (**C**) Correlation of THY1 gene expression with the infiltration level of immune cells in UCEC. (**D**) Correlation of COL1A1 gene expression with the infiltration level of immune cells in PDGFRA. The statistical significance is indicated by a p-value < 0.05

Following, we employed the TIMER2.0 database to explore potential links between the mRNA expression levels of COL1A1, ITGB1, THY1, and PDGFRA and immune cell infiltration in UCEC. An immune cell infiltration atlas was generated, encompassing CD4 + T cells, B cells, macrophages, and CD8 + T cells (Fig. 7A-D). The heatmap displayed in Fig. 7 depicts the associations between these immune cell types infiltrating the tumor cells across each sample. Overall, our analysis revealed significant correlations (p-value < 0.05) between the mRNA expression of COL1A1, ITGB1, THY1, and PDGFRA and the presence of CD4 + T cells, B cells, macrophages, and CD8 + T cells in UCEC (Fig. 7A-D).

### Gene enrichment and drug sensitivity analyses of immune-related genes

**Fig. 8:**
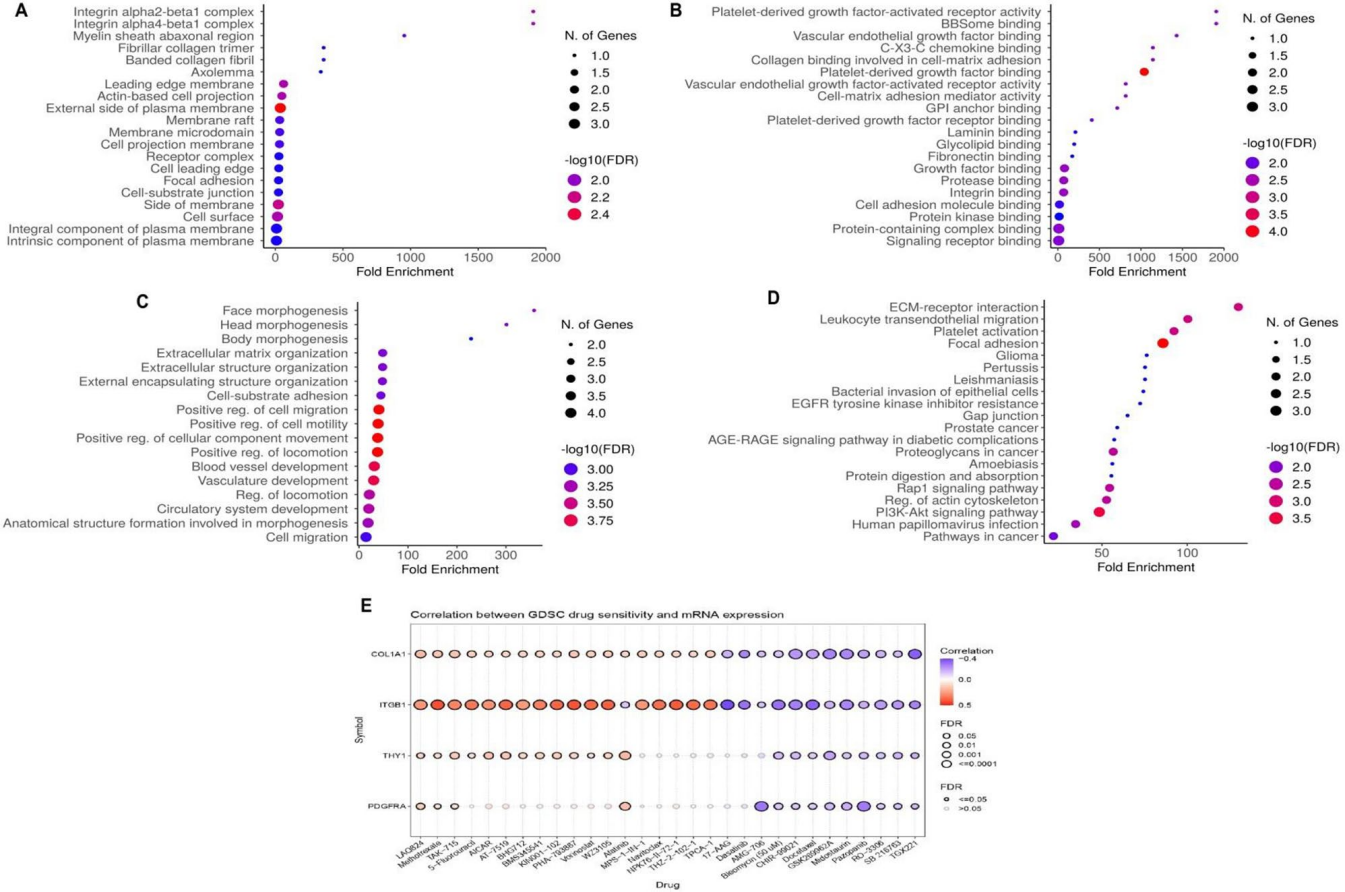
Gene Ontology (GO), Kyoto Encyclopedia of Genes and Genomes (KEGG), and Drug Sensitivity Analysis Associated with COL1A1, ITGB1, THY1, and PDGFRA Genes. (**A**) Cellular Component (CC) terms. (**B**) Molecular Function (MF) terms. (**C**) Biological Process (BP) terms. (**D**) KEGG pathway terms. (**E**) Drug sensitivity analysis of COL1A1, ITGB1, THY1, and PDGFRA genes. The statistical significance is indicated by a p-value < 0.05

Functional and pathway enrichment analyses of COL1A1, ITGB1, THY1, and PDGFRA genes were conducted using the DAVID tool. Gene Ontology (GO) analysis revealed their involvement in various cellular components (CC), molecular functions (MF), and biological processes (BP), particularly emphasizing extracellular matrix organization (Fig. 8A-C). Additionally, the top Kyoto Encyclopedia of Genes and Genomes (KEGG) pathways associated with COL1A1, ITGB1, THY1, and PDGFRA genes included pathways such as “ECM receptor interaction, leukocyte transendothelial migration, platelet activation, focal adhesion, and glioma” among others (Fig. 8D). These findings illuminate the diverse biological functions and pathways in which these genes participate, offering valuable insights into their potential roles in UCEC pathogenesis. Additionally, drug sensitivity analysis of COL1A1, ITGB1, THY1, and PDGFRA genes was conducted using the GSCA database. The results unveiled associations between these genes and the sensitivity to key drugs (Fig. 8E), highlighting potential therapeutic targets and avenues for personalized treatment strategies in UCEC.

### Inducing overexpression of COL1A1 and performing functional assays

**Fig. 9:**
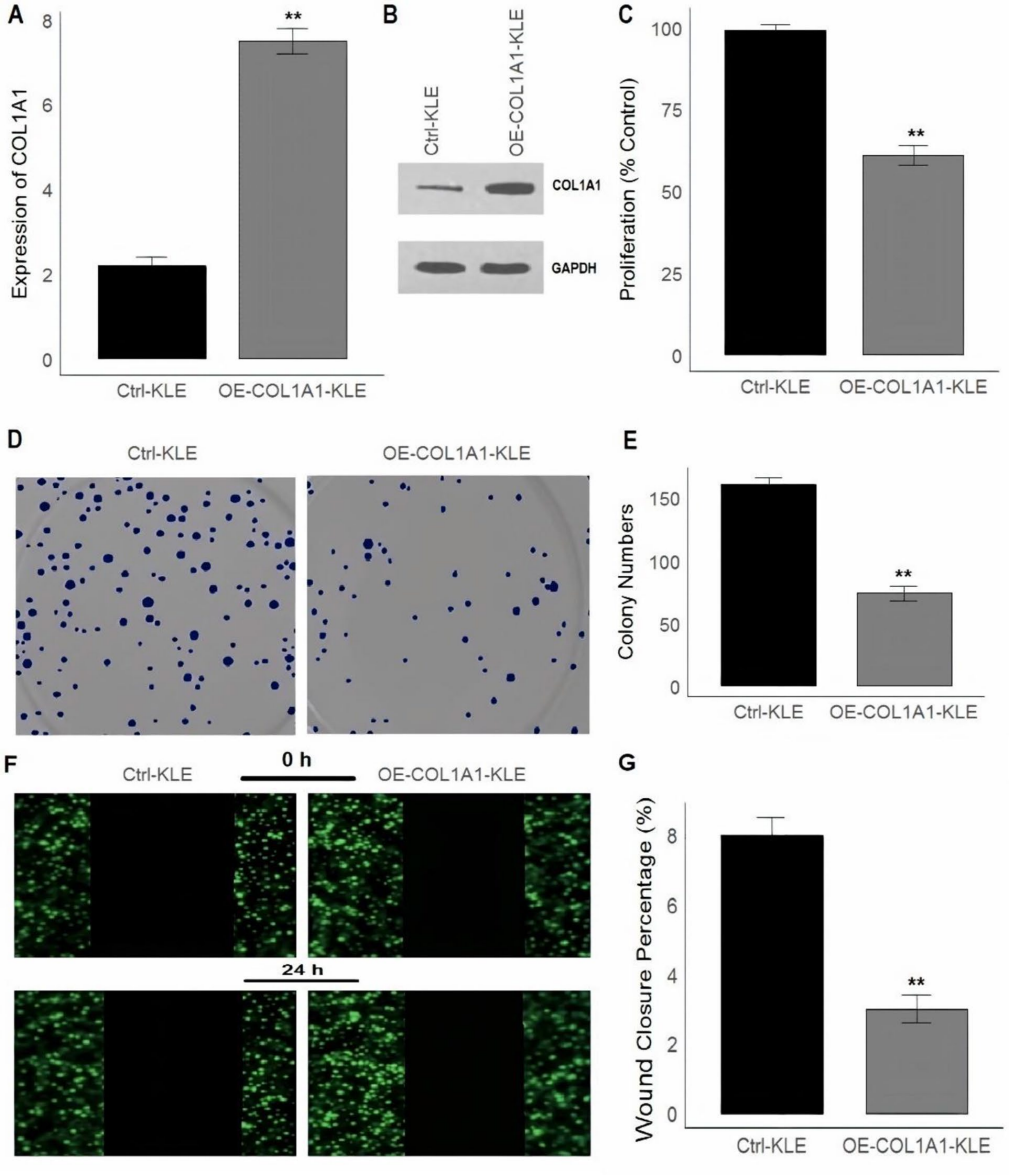
Overexpression of COL1A1 in KLE Cells Inhibits Proliferation, Colony Formation, and Migration. **(A**) Bar graph showing the relative expression levels of COL1A1 in Ctrl-KLE and OE-COL1A1-KLE cells. (**B**) Western blot bands of COL1A1 and GAPDH in Ctrl-KLE and OE-COL1A1-KLE cells. (**C**) Proliferation of KLE cells as a percentage of the control, demonstrating a significant reduction in the OE-COL1A1-KLE cells compared to the control. (**D**) Representative images from a colony formation assay, where the OE-COL1A1-KLE cells show fewer and smaller colonies compared to Ctrl-KLE cells. (**E**) Quantification of the colony numbers from the colony formation assay, showing a significant decrease in colony numbers in the OE-COL1A1-KLE cells. (**F**) Images from a wound healing assay at 0 and 24 h, showing reduced wound closure in OE-COL1A1-KLE cells compared to Ctrl-KLE cells. (**G**) Quantification of wound closure percentage at 24 h, highlighting a significant reduction in wound closure in the OE-COL1A1-KLE group. P**< 0.01

**Fig. 10:**
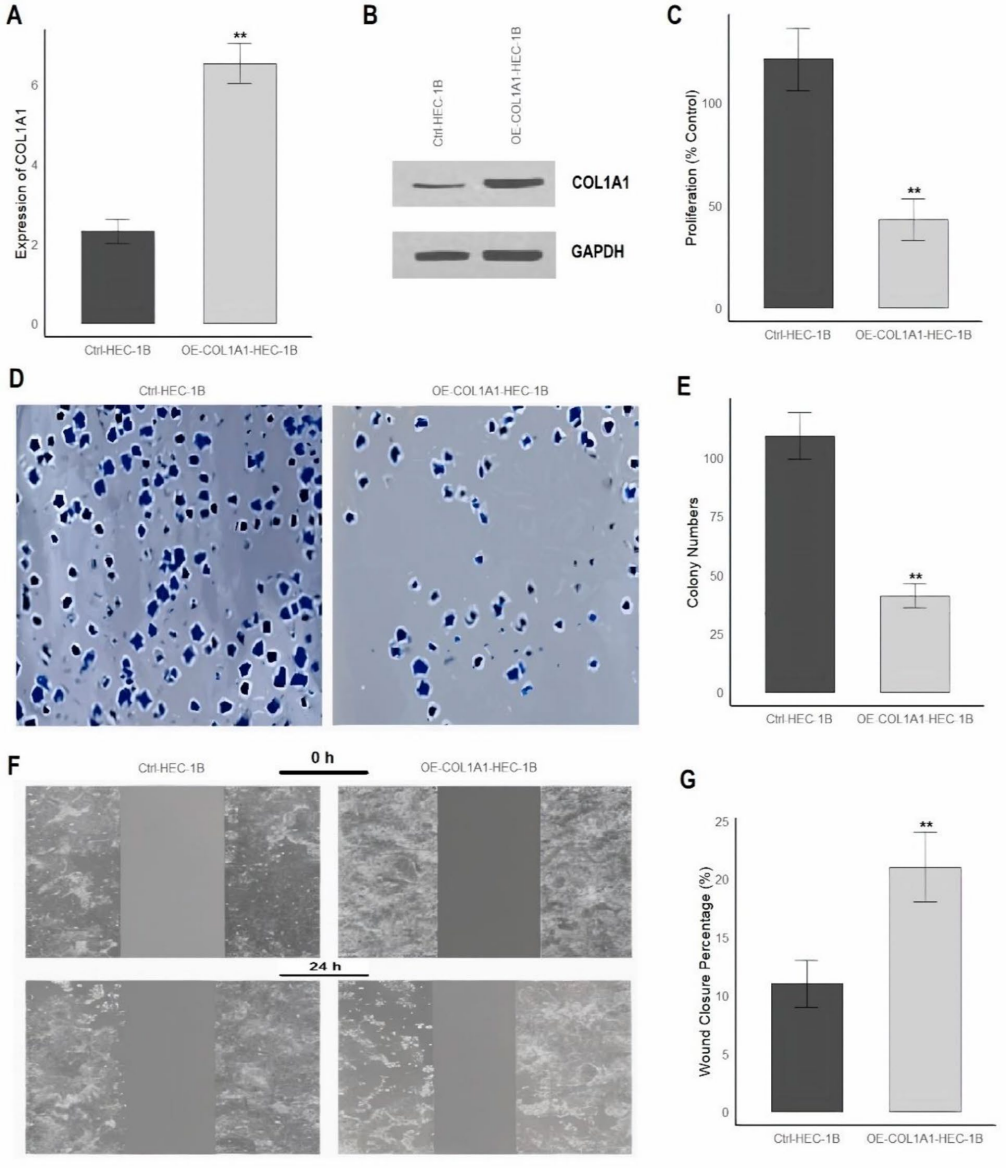
Overexpression of COL1A1 in HEC-1B Cells Inhibits Proliferation, Colony Formation, and Migration. **(A**) Bar graph showing the relative expression levels of COL1A1 in Ctrl-HEC-1B and OE-COL1A1-HEC-1B cells. (**B**) Western blot bands of COL1A1 and GAPDH in Ctrl-HEC-1B and OE-COL1A1-HEC-1B cells. (**C**) Proliferation of Ctrl-HEC-1B as a percentage of the control, demonstrating a significant reduction in the OE-COL1A1-HEC-1B cells compared to the control. (**D**) Representative images from a colony formation assay, where the OE-COL1A1-HEC-1B cells show fewer and smaller colonies compared to Ctrl-HEC-1B cells. (**E**) Quantification of the colony numbers from the colony formation assay, showing a significant decrease in colony numbers in the OE-COL1A1-KLE cells. (**F**) Images from a wound healing assay at 0 and 24 h, showing reduced wound closure in OE-COL1A1-HEC-1B cells compared to Ctrl-HEC-1B cells. (**G**) Quantification of wound closure percentage at 24 h, highlighting a significant reduction in wound closure in the OE-COL1A1-KLE group. P**< 0.01

Finally, the overexpression of COL1A1 was induced in KLE and HEC-1B cells to analyze its impact on various cellular behaviors. In Figs. 9A-B and 10A-B, the bar graph and Western blot images (Supplementary data Fig. 1) revealed that the OE-COL1A1-KLE and OE-COL1A1-HEC-1B cells exhibited significantly higher expression levels of the COL1A1 gene compared to the control Ctrl-KLE and Ctrl-HEC-1B cells. After confirming COL1A1 overexpression, Figs. 9C and 10C demonstrated a substantial reduction in cell proliferation in OE-COL1A1-KLE and OE-COL1A1-HEC-1B cells relative to Ctrl-KLE and Ctrl-HEC-1B cells. Figures 9D-E and 10D-E showed a clear reduction in the number and size of colonies formed by OE-COL1A1-KLE and OE-COL1A1-HEC-1B cells compared to Ctrl-KLE and Ctrl-HEC-1B cells. Additionally, cell migration, an important factor in cancer metastasis, was assessed in Figs. 9F-G and 10F-G, where wound healing assays at 0 and 24 h revealed that Ctrl-KLE and Ctrl-HEC-1B cells demonstrated significant wound closure, indicating robust migratory activity. In contrast, OE-COL1A1-KLE and OE-COL1A1-HEC-1B cells showed markedly reduced wound closure, suggesting that COL1A1 overexpression hindered their ability to migrate effectively. This observation was quantitatively supported by Figs. 9G and 10G, where results showed significantly lower wound closure percentages in OE-COL1A1-KLE and OE-COL1A1-HEC-1B cells, reinforcing the impaired migratory capacity.

## Discussion

The immune system plays a critical role in preventing UCEC by detecting and eliminating abnormal cells [29, 30]. However, in UCEC, dysregulation of COL1A1, ITGB1, THY1, and PDGFRA may compromise immune function, enabling tumor progression. COL1A1 promotes excessive collagen production, creating a dense ECM that blocks immune cell infiltration [8]. ITGB1 disrupts immune cell adhesion and migration, impairing immune surveillance [31]. THY1 enhances CAF activity, leading to an immunosuppressive environment [32, 33], while PDGFRA fosters fibrosis, further hindering immune cell access to the tumor [34]. Looking at the diverse roles, this study comprehensively investigated COL1A1, ITGB1, THY1, and PDGFRA genes in UCEC using multi-level methodology.

Our investigation revealed that both the transcriptional and translational levels of COL1A1, ITGB1, THY1, and PDGFRA were down-regulated in UCEC patients. This decreased expression was associated with different stages of cancer progression in UCEC. Survival analysis indicated that lower mRNA expression of these genes was correlated with poorer overall survival rates among UCEC patients. These findings suggest the potential of COL1A1, ITGB1, THY1, and PDGFRA as potential biomarkers for the diagnosis and prognosis of UCEC. However, further studies are needed to confirm their clinical applicability in guiding treatment strategies.

When COL1A1, ITGB1, THY1, and PDGFRA are down-regulated, they contribute to a cascade of molecular events that profoundly impact cancer development and progression. COL1A1, a key component of the ECM, provides structural integrity to tissues. Down-regulation of COL1A1 alters the ECM composition, compromising cell adhesion, migration, and signaling processes crucial for maintaining tissue architecture [35, 36]. This disruption facilitates tumor invasion and metastasis. Similarly, ITGB1, a cell surface receptor involved in cell adhesion and migration, plays a pivotal role in tumor progression. Decreased ITGB1 expression impairs cell-ECM interactions and signaling pathways essential for cell proliferation and survival, promoting tumor cell motility, invasion, and metastasis [37].

THY1, a glycoprotein regulating cell-cell and cell-matrix interactions, is down-regulated in various cancers [38, 39]. Reduced THY1 expression disrupts cell adhesion and communication, altering tissue architecture and ECM remodeling [40, 41]. This dysregulation correlates with increased tumor aggressiveness, metastasis, and therapy resistance [42]. Additionally, PDGFRA, a receptor tyrosine kinase essential for cell proliferation and survival, exhibits decreased expression in cancer [43, 44]. Lower PDGFRA level impair signaling pathways regulating tumor growth and progression, leading to reduced angiogenesis, invasion, and metastasis [45, 46]. Collectively, the down-regulation of wound healing genes, including COL1A1, ITGB1, THY1, and PDGFRA contributes to the disruption of normal tissue architecture, compromised cell adhesion and migration, and dysregulated signaling pathways involved in proliferation and survival. These molecular alterations create an environment conducive to cancer initiation, progression, and metastasis, highlighting the critical role of wound-healing genes in cancer pathogenesis.

The regulatory mechanisms dictating the expression of COL1A1, ITGB1, THY1, and PDGFRA in UCEC were comprehensively explored through miRNA prediction and validation. Our findings uncovered a network of 222 miRNAs implicated in modulating the expression of these genes, with has-mir-16-5p and has-mir-128-3p emerging as significant regulators targeting all four genes simultaneously. This observation aligns with previous studies indicating the regulatory roles of miRNAs in cancer progression [47, 48]. The down-regulation of has-mir-16-5p and has-mir-128-3p miRNAs in UCEC tissues further strengthens their potential as key modulators of gene expression dysregulation in cancer. Moreover, immune correlation analysis revealed intricate associations between the mRNA expression levels of COL1A1, ITGB1, THY1, and PDGFRA and immune cell infiltration in UCEC. The significant correlations observed between these genes and various immune cell types, including CD4 + T cells, B cells, macrophages, and CD8 + T cells, emphasize the immunomodulatory roles of these genes in shaping the tumor microenvironment. This finding corroborates previous research highlighting the crosstalk between cancer cells and immune cells, influencing tumor progression and therapeutic responses [49–51]. Functional and pathway enrichment analyses provided insights into the biological functions and pathways associated with COL1A1, ITGB1, THY1, and PDGFRA genes. The involvement of these genes in processes such as extracellular matrix organization and pathways like ECM receptor interaction and focal adhesion underscores their crucial roles in UCEC pathogenesis.

Despite the comprehensive nature of this study, a few limitations need to be addressed. Firstly, the relatively small sample size for certain analyses, particularly in survival and cancer stage correlation studies, may have limited the statistical power and generalizability of the findings. Additionally, while the in silico and in vitro approaches provided valuable insights into the roles of COL1A1, ITGB1, THY1, and PDGFRA in UCEC, the results still require validation in larger clinical cohorts to confirm their clinical relevance. Furthermore, while miRNA prediction and immune correlation analyses were insightful, the specific mechanisms underlying the regulation of these genes by miRNAs and their exact impact on immune cell infiltration require further experimental validation. Finally, the lack of functional in vivo studies examining the direct therapeutic targeting of these genes limits the ability to translate these findings into potential clinical applications.

## Conclusion

In summary, our investigation into the role of immune-related genes, including COL1A1, ITGB1, THY1, and PDGFRA, in UCEC sheds light on their multifaceted contributions to cancer development and progression. Down-regulation of these genes, as observed in our study, disrupts crucial cellular processes such as cell adhesion, migration, and ECM remodeling, fostering a tumor-promoting microenvironment conducive to UCEC progression. These findings emphasize the potential utility of COL1A1, ITGB1, THY1, and PDGFRA as diagnostic biomarkers, prognostic indicators, and therapeutic targets in UCEC management. Further exploration of their regulatory mechanisms, immune correlations, and functional pathways offers promising avenues for personalized treatment strategies and improved patient outcomes in UCEC.

## Electronic supplementary material

Below is the link to the electronic supplementary material.

Supplementary Material 1

## Data Availability

Any type of the data will be provided by the corresponding author.
