# Supplementary figures and images for: COL1A1, ITGB1, THY1, and PDGFRA: key immune-related genes in uterine corpus endometrial carcinoma with prognostic and therapeutic implications
Source: Hereditas. 2025 Aug 15;162:159. doi: 10.1186/s41065-025-00448-x (PMC12357369; doi:10.1186/s41065-025-00448-x)

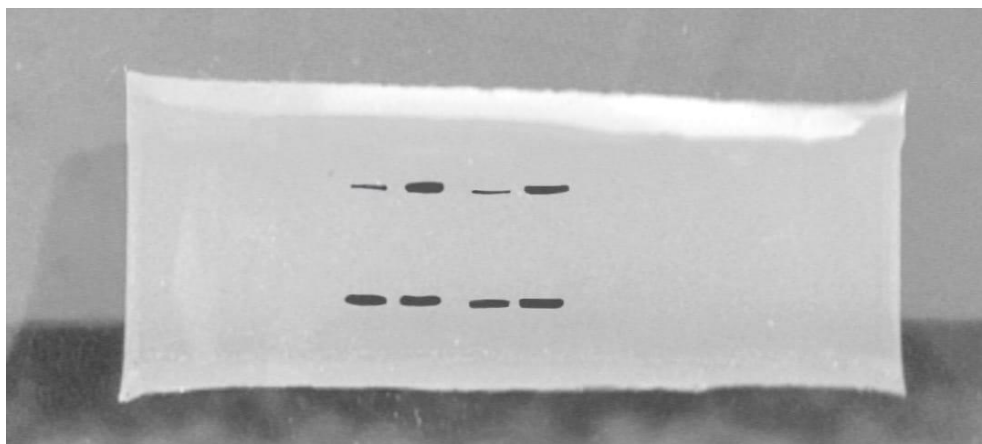

**Supplementary data Figure 1: Uncut Western blot bands of GAPDH and COL1A1.**

Supplement: Supplementary file 1 — Supplementary Material 1 [file 41065_2025_448_MOESM1_ESM.pdf]
